# Survey Protocols, Response Rates, and Representation of Underserved Patients: A Randomized Clinical Trial

**DOI:** 10.1001/jamahealthforum.2023.4929

**Published:** 2024-01-19

**Authors:** Marc N. Elliott, Julie A. Brown, Katrin Hambarsoomian, Layla Parast, Megan K. Beckett, William G. Lehrman, Laura A. Giordano, Elizabeth H. Goldstein, Paul D. Cleary

**Affiliations:** 1 RAND Corporation, Santa Monica, California; 2 University of Texas, Austin; 3 Centers for Medicare & Medicaid Services, Baltimore, Maryland; 4 Health Services Advisory Group, Phoenix, Arizona; 5 Yale School of Public Health, New Haven, Connecticut

## Abstract

**Importance:**

Surveys often underrepresent certain patients, such as underserved patients. Methods that improve their response rates (RRs) would help patient surveys better represent their experiences and assess equity and equity-targeted quality improvement efforts.

**Objective:**

To estimate the effect of adding an initial web mode to existing Hospital Consumer Assessment of Healthcare Providers and Systems (HCAHPS) survey protocols and extending the fielding period on RR and representativeness of underserved patient groups.

**Design, Setting, and Participants:**

This randomized clinical trial included 36 001 patients discharged from 46 US hospitals from May through December 2021. Data analysis was performed from May 2022 to September 2023.

**Exposures:**

Patients were randomized to 1 of 6 survey protocols: 3 standard HCAHPS protocols (mail only, phone only, mail-phone) plus 3 web-enhanced protocols (web-mail, web-phone, web-mail-phone).

**Main Outcomes and Measures:**

RR and number of respondents per 100 survey attempts (yield) were calculated and compared for each of the 6 survey protocols, overall, and by patient age, service line, sex, and race and ethnicity.

**Results:**

A total of 34 335 patients (median age range, 55-59 years; 59.3% female individuals and 40.7% male individuals) were eligible and included in the study. Of the respondents, 6.9% were Asian American or Native Hawaiian or Other Pacific Islander, 0.7% were American Indian or Alaska Native, 11.5% were Black, 17.4% were Hispanic, 61.0% were White, and 2.6% were multiracial. Of the 6 protocols, RRs were highest in web-mail-phone (36.5%), intermediate for the 3 two-mode survey protocols (mail-phone, web-mail, web-phone, 30.3%-31.1%), and lowest for the 2 single-mode protocols (mail only, phone only, 22.1%-24.3%). Web-mail-phone resulted in the highest yield for 3 racial and ethnic groups (Black, Hispanic, and White patients) and second highest for another (multiracial patients). Otherwise, the highest or second highest yield was almost always a 2-mode protocol. Mail only was the lowest-yield protocol for Black, Hispanic, and multiracial patients and phone-only was the lowest-yield protocol for White patients; these 2 protocols tied for lowest-yield for Asian American or Native Hawaiian or Other Pacific Islander patients. Gains from multimode approaches were often 2 to 3 times as large for Asian American or Native Hawaiian or Other Pacific Islander, Black, Hispanic, and multiracial patients as for White patients. Web-mail-phone had the highest RR for 6 of 8 age groups and 4 of 5 combinations of service line and sex.

**Conclusions and Relevance:**

In this randomized clinical trial, web-first multimode survey protocols significantly improved the RR and representativeness of patient surveys. The best-performing protocol based on RR and representativeness was web-mail-phone. Web-phone performed well for young and diverse patient populations, and web-mail for older and less diverse patient populations. The US Centers for Medicare & Medicaid Services will allow hospitals to use the web-mail, web-phone, and web-mail-phone protocols for HCAHPS administration beginning in 2025.

## Key Points

**Question:**

Does web enhancement of existing Hospital Consumer Assessment of Healthcare Providers and Systems survey modes improve response rates and representation of underserved patients?

**Findings:**

This randomized clinical trial involving 36 001 patients from 46 US general hospitals found that a 3-phase survey protocol (web-mail-phone) resulted in the highest response rate and patient representativeness. Two-phase survey protocols were almost always the second-best approach, especially web-phone for younger, more diverse patient populations and web-mail for older, less diverse patient populations.

**Meaning:**

Web-enhanced multimode survey protocols will likely improve response rates and representation of vulnerable patient groups.

## Teaser

This randomized clinical trial estimates the effect of adding an initial Web mode to patient experience survey protocols and extending the fielding period on response rates and representation of underserved individuals.

## Introduction

The Hospital Consumer Assessment of Healthcare Providers and Systems (HCAHPS) survey is used to measure and publicly report hospital patient care experiences and determine part of the value-based incentive payments in the Hospital Value-Based Purchasing program. In 2022, HCAHPS scores accounted for 25% of incentive payments.^1^ Response rates (RRs) to all surveys, including patient experience surveys, have been falling for many years.^2,3,4,5^ Low RRs can jeopardize a hospital’s eligibility for incentive payments, which requires at least 100 completed surveys.

In general, surveys, including patient experience surveys, have lower RRs for adults who are Black, Hispanic, and younger; this may also hold true for Asian American and Native Hawaiian or Other Pacific Islander adults with Medicare, for whom findings are less clear.^6,7,8,9,10^ Methods that improve RRs for these groups are critical to ensuring that responses to patient experience surveys appropriately represent the care experiences of all patients and adequately measure health equity and equity-targeted quality improvement efforts. Moreover, any effort that improves RRs for these groups is likely to improve overall representativeness.

Since the national administration of HCAHPS began in 2006, hospitals have been allowed to choose among several data collection protocols: mail only, phone only, mixed mode (mail with phone follow-up of nonrespondents), and active interactive voice response.^11^ Previous research on HCAHPS surveys showed that mixed mode consistently yields higher RRs than the single-mode protocols.^12,13^ Sequential multimode approaches increase RRs and representativeness because patients vary in their preferred mode; offering 2 or more modes sequentially increases the likelihood that patients can respond in their preferred mode. Herein, we investigate the effects of 3 web-first protocols (web-mail, web-phone, and web-mail-phone) relative to existing HCAHPS protocols.

HCAHPS currently allows patients to respond up to 42 days after the first contact attempt. Longer data collection periods have several potential benefits, including facilitating sequential multimode protocols and possibly increasing RRs and representativeness.^14^ To allow adequate time for multimode protocols, we conducted this experiment with a 49-day rather than a 42-day data collection period. We calculated the RR gains from the final 7 days and compared respondents in the last 7 days to those in the first 42 days.

## Methods

A randomized study of 6 different survey administration protocols was conducted from May (with April discharges) through December 2021. A sample of 36 001 discharges was selected from a nationally representative sample of 46 short-term acute care hospitals listed in the 2021 American Hospital Association Annual Survey of Hospitals with at least 1200 annual inpatient stays. Hospitals were recruited from among all hospitals that collected HCAHPS data, had their data reported on the Centers for Medicare & Medicaid Services (CMS) Hospital Compare website (now Care Compare), and had sufficient discharges to provide a sample for the study and regular HCAHPS implementation (described in eMethods in Supplement 1). Each hospital provided a monthly frame of discharged patients whose eligibility for HCAHPS could be assessed using administrative records. Eligibility criteria included being at least age 18 years at admission, having had at least 1 inpatient overnight stay with a nonpsychiatric principal diagnosis, and being discharged alive.

A random sample was drawn from each hospital’s pool of eligible patients prior to the sample draw for regular HCAHPS implementation (sample size determination and patient flow are described in eMethods in Supplement 1). Within each hospital, each sampled patient was randomly assigned to 1 of 6 data collection protocols (eMethods in Supplement 1). Probabilities of randomization to each protocol differed, with probabilities inversely proportionate to the anticipated RR for each protocol, to obtain similar numbers of completed surveys for each protocol. A single organization collected data at all hospitals using the standard HCAHPS vendor protocols.^15^ HCAHPS survey administration began 2 to 49 days after the patient was discharged from the hospital and was completed within 49 days after first contact attempt. Table 1 shows the schedule of contacts. In the mail-only protocol, a second survey was mailed if there was no response by 21 days after the first mailing. The phone-only protocol entailed 5 different phone call attempts, if needed. Call attempts were made at different times of the day, on different days of the week, and in different weeks. The first contact for mixed-mode survey administration was by mail. If the mailed survey was not completed and returned within 28 days, follow-up phone contact was attempted using the same protocol as for the phone-only mode. The web-first version of these 3 modes began with an email notification of the web survey that contained a clickable, personalized link to the online survey, which was mobile optimized.

**Table 1.** Hospital Consumer Assessment of Healthcare Providers and Systems 2021 Mode Experiment Schedule of Contacts

Day	Mail only	Phone only	Mixed mode	Web-mail	Web-phone	Web-mail-phone
1	Mail first survey	Begin phone calls	Mail survey	Email first invitation	Email first invitation	Email first invitation
3				Email second invitation		
4					Email second invitation	Email second invitation
6				Email third invitation		Mail survey
7					Email third invitation	
8				Mail first survey		
10					Begin phone calls	
21	Mail second survey					
28			Begin phone calls			Begin phone calls
30				Mail second survey		
49	End data collection	End data collection	End data collection	End data collection	End data collection	End data collection

A patient with an email address available (EMA) who was randomized to a web-first protocol experienced the full protocol. A patient with no email address available (NEMA) who was randomized to a web-first protocol (eg, web-phone) experienced a delayed traditional mode (eg, NEMA web-phone-only mode was a delayed phone-only protocol). In such a case, the follow-up modes (mail, phone, or both) for NEMA patients happened at the same time as for EMA patients, an approach designed to increase the efficiency and feasibility of the protocols for HCAHPS survey vendors. EMA and NEMA patients randomized to nonweb protocols had identical experiences.

Because age, sex, and service line are collected administratively in HCAHPS and are available for both respondents and nonrespondents, RRs by these characteristics can be directly calculated. HCAHPS collects self-reported race and ethnicity; this information is only available for respondents. The HCAHPS survey provides the following race and ethnicity categories: American Indian or Alaska Native; Asian American and Native Hawaiian or Other Pacific Islander; Black or African American (hereafter, Black); Spanish, Hispanic, or Latino origin or descent (hereafter, Hispanic); White; and multiracial.

RRs were calculated as the number of completed surveys divided by the number of eligible fielded cases (or discharged patients), with ineligible cases removed from the denominator. Unstandardized RRs are invalid for comparisons across survey administration protocols because they understate ineligible patients and therefore overstate RRs for protocols without a phone component relative to those with a phone component. The phone mode provides a more accurate estimate of the true ineligibility rate, as ineligibility that would not be established otherwise is often detected by phone (eg, if a patient has died, a mailed survey will often simply not be returned, but phone contact may reach a family member who can provide this information).^12^ We therefore standardized the RR calculations across the 6 arms, relying on randomization, by using the average ineligible rate of 7.1% for each arm, based not on the ineligibility rate observed in each arm but on the mean observed rate of ineligibility in the 4 arms that include phone (phone only, web-phone, mail-phone, web-mail-phone).

Yield is the number of completed surveys divided by all fielded cases and calculated as the number of completions received per 100 surveys attempted, including ineligible patients. While yield understates RRs, it is comparable across protocols that differ in how well they detect ineligible patients (eg, phone only vs mail only) in a randomized experiment. Randomization allows comparison of yield across protocols to learn how well protocols represent characteristics that are only known for respondents, such as race and ethnicity for HCAHPS.

RRs and yield were compared to a reference mail-only mode using logistic regression. The characteristics of early (0-42 days) and late (43-49 days) responders were compared using logistic regression. All confidence intervals were 95% CIs based on normal approximations of the binomial. Two-tailed *t* tests with a significance level of .05 were used.

This study was approved by the RAND Corporation Institutional Review Board; the requirement for written informed consent was waived because the study met the criteria detailed in the Privacy Rule.^16^ We followed the Consolidated Standards of Reporting Trials (CONSORT) reporting guideline.^17^ Analyses were performed using SAS, version 9.4 (SAS Institute).

## Results

Of the 36 001 patients sampled for the mode experiment, 1666 (4.6%) were ineligible because they were unable to complete the survey due to a physical, mental, or language barrier or because they had died; thus, the study included 34 335 eligible patients (median age range, 55-59 years; 59.3% female individuals and 40.7% male individuals). Of the respondents, 0.7% were American Indian or Alaska Native, 6.9% were Asian American or Native Hawaiian or Other Pacific Islander, 11.5% were Black, 17.4% were Hispanic, 61.0% were White, and 2.6% were multiracial. In Supplement 1, eTable 1 compares the hospital characteristics of the 46 hospitals in the mode experiment with all hospitals participating in HCAHPS in 2021; eTable 2 shows the corresponding patient characteristics by survey administration protocol. Overall, largely because of the minimum sample size requirement, the hospitals participating in the mode experiment were larger (91% had 200 or more beds vs 28% of all HCAHPS hospitals), more often for profit (26% vs 16%), and were located more often in the South (46% vs 23%) and less often in the Midwest (17% vs 30%) or the Northeast (11% vs 28%).

Compared to patients in all 2021 HCAHPS-participating hospitals, the 34 335 eligible patients sampled in the mode experiment were more often maternity patients (19% vs 16%) rather than medical patients (55% vs 60%). Patients in mode experiment hospitals were more racially and ethnically diverse than hospitals overall (7% vs 3% Asian American or Native Hawaiian or Other Pacific Islander, 12% vs 8% Black, 17% vs 11% Hispanic). The percentage of sampled patients with an email address varied by hospital, ranging from 11% to 94%, with a hospital-level average of 64% and an overall rate of 63% (results not shown). Overall RRs also varied by hospital, ranging from 14% to 40% (results not shown).

**Figure 1.**
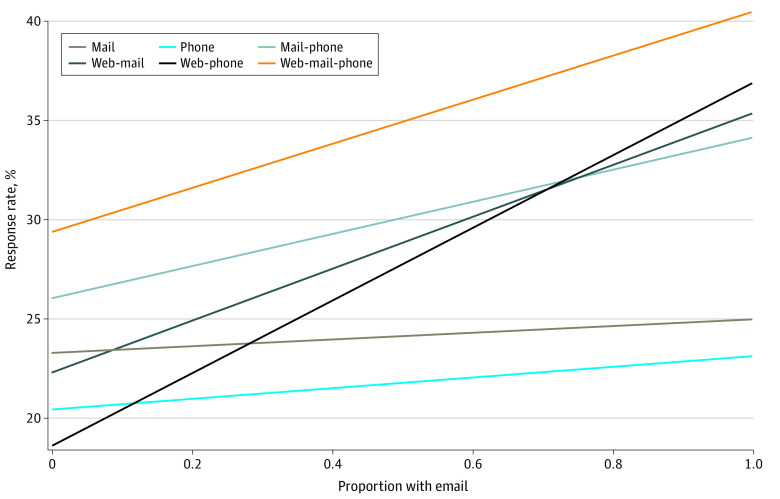
Predicted Response Rates by Proportion With Email Addresses, by Survey Administration Protocol

Table 2 presents patient-level standardized RRs (SRRs) overall and by protocol, pooled and stratified by EMA/NEMA; modes of completion appear in eTable 3 in Supplement 1. The pooled SRRs among the eligible patients were 22.1% for phone only, 24.3% for mail only, 30.3% for web-phone, 30.6% for web-mail, 31.1% for mail-phone, and 36.5% for web-mail-phone. As shown in the last column of Table 2, having an email address (EMA) was generally associated with higher RRs, particularly in web-first modes (by 11.1 to 18.3 percentage points [pp], compared to 1.7 to 8.0 pp in nonweb modes). Figure 1 plots estimates from Table 2 to show that predicted RRs increase as the percentage of EMA increases at the hospital level, with steeper increases in protocols with a web component. Within the EMA sample, web-first protocols added to SRRs 10.4 pp (or 42%) for mail only, 13.8 pp (or 60%) for phone only, and 6.3 pp (or 18%) for mail-phone.

**Table 2.** Standardized Response Rates by Survey Administration Protocol (N = 36 001)

Survey administration protocol (No. fielded)	Standardized response rate, % (95% CI)^a^			EMA − NEMA (difference)
	Pooled	Email availability		
		EMA^b^	NEMA	
**HCAHPS legacy modes**				
Mail only (n = 7010)	24.3 (23.3 to 25.4)	24.9 (23.6 to 26.2)	23.2 (21.5 to 25.0)	1.7 (−0.5 to 3.9)
Phone only (n = 5979)	22.1 (21.1 to 23.2)	23.1 (21.7 to 24.5)	20.4 (18.6 to 22.2)	2.7 (0.5 to 5.0)
Mail-phone (n = 5009)	31.1 (29.8 to 32.5)	34.1 (32.4 to 35.8)	26.1 (24.0 to 28.1)	8.0 (5.3 to 10.7)
**Web-first protocols**				
Web-mail (n = 7008)	30.6 (29.5 to 31.8)	35.3 (33.8 to 36.8)	22.3 (20.6 to 24.0)	13.0 (10.8 to 15.3)
Web-phone (n = 5997)	30.3 (29.1 to 31.5)	36.9 (35.3 to 38.5)	18.6 (16.9 to 20.3)	18.3 (16.0 to 20.6)
Web-mail-phone (n = 4998)	36.5 (35.1 to 37.9)	40.4 (38.7 to 42.2)	29.4 (27.2 to 31.5)	11.1 (8.3 to 13.9)

Abbreviations: EMA, email address available; HCAHPS, Hospital Consumer Assessment of Healthcare Providers and Systems survey; NEMA, no email address available.

^a^ The 95% CIs are based on normal approximations of the binomial.

^b^ Percentage of fielded cases with email addresses: mail only, 63.4%; phone only, 63.1%; mail-phone, 62.2%; web-mail, 63.1%; web-phone, 62.8%; web-mail-phone, 63.0%.

### RRs by Respondent Characteristics

**Figure 2.**
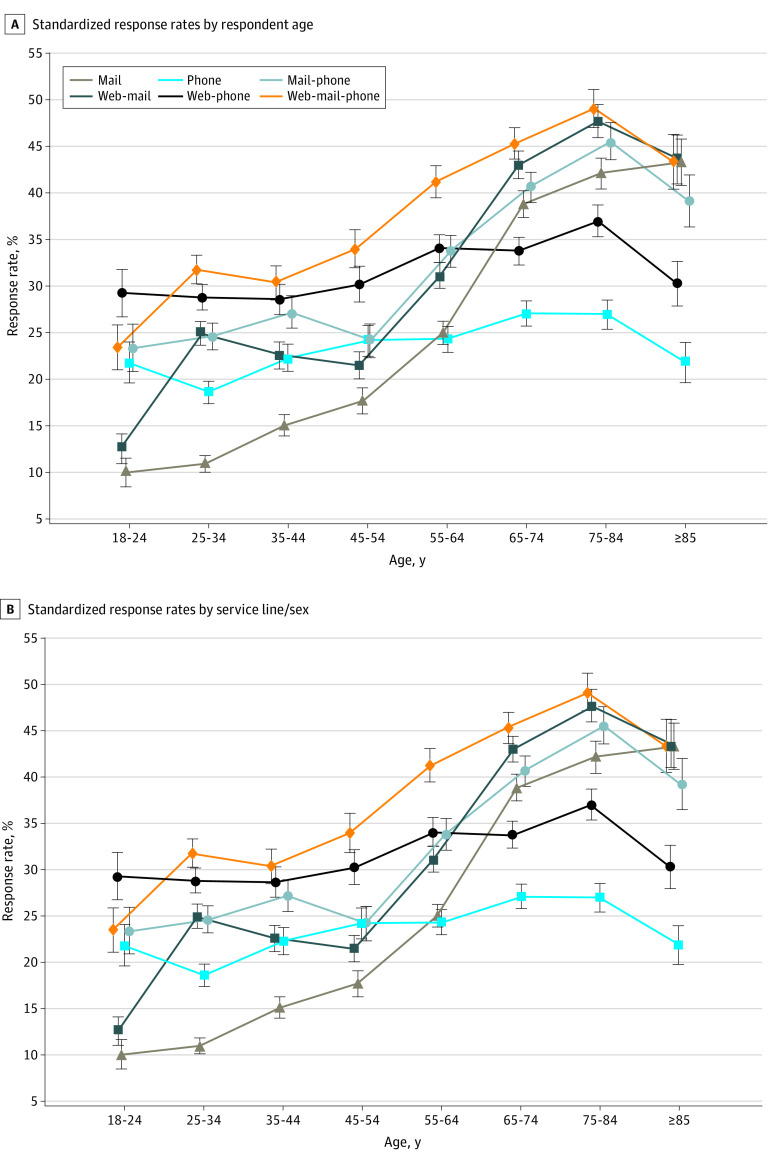
Standardized Response Rates by Respondent Age and Service Line/Sex Error bars indicate 95% CIs.

As shown in Figure 2A (and eTables 4 and 6 in Supplement 1), the web-mail-phone protocol had the highest SRR for 6 of 8 age groups (all but ages 18-24 years and age ≥85 years), the second highest RR for the youngest, and the third highest RR for the oldest age group. Otherwise, the highest or second highest SRR was almost always a 2-mode protocol: web-phone for ages 25 to 64 years and web-mail for ages 65 years and older. For all ages, a single-mode protocol had the lowest RR: mail only had the lowest SRR for ages 18 to 54 years, and phone only had lowest SRRs for 55 years and older (though mail only was statistically indistinguishable from phone only for ages 55-64 years).

As shown in Figure 2B (and eTables 5 and 7 in Supplement 1), web-mail-phone had the highest SRR for 4 of 5 combinations of service line and sex and tied for the highest SRR for the other group (maternity patients). Otherwise, the second highest SRR was always a 2-mode protocol; among the 2-mode protocols, mail-phone and web-mail did especially well for surgical patients, mail-phone especially well for medical patients, and web-phone especially well for maternity patients. Mail only had the lowest SRR for maternity (12.3%), and phone only the lowest for other service lines (ranging from 19.4% for medical-female to 28.7% for surgical-female).

### Yield and Race and Ethnicity

Table 3 shows that web-mail-phone had the highest yield for 3 of 5 racial and ethnic groups (Black, Hispanic, and White patients). Otherwise, the highest or second highest yield was almost always a 2-mode protocol. Mail only was the lowest-yield protocol for Black, Hispanic, and multiracial patients; phone only was the lowest-yield protocol for White patients; and these protocols tied as the lowest-yield protocols for Asian American or Native Hawaiian or Other Pacific Islander patients. The gains from multimode approaches are often 2 to 3 times as large for Asian American or Native Hawaiian or Other Pacific Islander, Black, Hispanic, and multiracial patients as for White patients (Table 3).

**Table 3.** Increase in Yield Compared to Mail Only by Survey Administration Protocol and Race and Ethnicity

Race and ethnicity^a^	Yield, % (95% CI)^b^	Increase in yield compared to mail only, % (95% CI)				
		Phone only	Mail-phone	Web-mail	Web-phone	Web-mail-phone
Asian American and Native Hawaiian or Other Pacific Islander	1.3 (1.0 to 1.6)	0 (−22 to 22)	69 (38 to 100)^c^^,^^d^	69 (43 to 96)^c^^,^^d^	38 (13 to 64)^c^	54 (24 to 84)^c^
Black	2.4 (2.0 to 2.8)	17 (−1 to 34)	33 (13 to 54)^c^	13 (−3 to 28)	29 (11 to 47)^c^	75 (52 to 98)^c^^,^^d^
Hispanic	3.0 (2.6 to 3.4)	37 (20 to 53)^c^	50 (31 to 69)^c^	53 (11 to 23)^c^	80 (61 to 99)^c^	90 (69 to 111)^c^^,^^d^
White	15.1 (14.3 to 15.9)	−31 (−36 to −26)^c^	12 (5 to 19)^c^	17 (11 to 23)^c^	1 (−5 to 7)	32 (24 to 39)^c^^,^^d^
Multiracial	0.2 (0.1 to 0.3)	450 (318 to 582)^c^^,^^d^	350 (219 to 481)^c^	50 (−14 to 114)^c^	450 (318 to 582)^c^^,^^d^	200 (93 to 307)^c^

^a^ American Indian and Alaska Native respondents are not included because the estimates are not reliably measured.

^b^ Reference: mail only.

^c^ Statistically significant (significance level, *P* < .05) for difference from mail-only via logistic regression. Confidence intervals are based on a normal approximation of the binomial.

^d^ Highest response rate for a given row.

### RRs and Length of Fielding Period

Days 43 to 49 increased the RR for all 6 protocols relative to the first 42 days, with the gain averaging 3 pp (results not shown). This gain was 1 pp in phone only, 2 pp in mail only, and 3 pp in each of the other 4 protocols. The largest gains from the extended fielding period were for underrepresented groups (eTable 8 in Supplement 1). Racial and ethnic minority respondents were 51% of last-week respondents vs 40% of earlier respondents (*P* < .001 for difference). Those preferring another language to English were 13% of last-week respondents vs 10% of earlier respondents (*P* < .001).

## Discussion

Survey RRs are usually lower for younger, racial and ethnic minority, and maternity patients. We find that sequential multimode survey protocols that include web, phone, or both and longer data collection periods can improve the representation of racial and ethnic groups in patient experience surveys such as HCAHPS. For HCAHPS, the gains from multimode approaches were often 2 to 3 times as large for Asian American or Native Hawaiian or Other Pacific Islander, Black, Hispanic, and multiracial people as for White people. There were also important gains in RRs for younger and maternity patients. The mail-only protocol had the lowest yield for Black, Hispanic, and multiracial patients; those aged 18 to 54 years; and maternity patients. The phone-only protocol had the lowest yield for White patients; those 55 years and older; and medical and surgical patients. The mail-only protocol had the lowest RR for patients aged 18 to 54 years and maternity patients. The phone-only mode had the lowest RR for patients 55 years and older and medical and surgical patients. Web-mail-phone had the highest RR for most groups. Among 2-mode protocols, web-phone was especially successful for maternity patients and patients aged 18 to 64 years, and web-mail was especially successful for surgical patients and those 65 years and older. Mail-phone was especially successful for medical patients.

While multimode approaches consistently outperform single-mode approaches, the most effective survey modes for a given hospital will depend on its patient population. We further found additional gains in RR by extending the survey response period from the current 42 days to 49 days, especially for racial and ethnic and linguistic minority patients. Later responses to patient experience surveys are also known to capture poorer experiences than earlier responses.^12,18,19^

Web-first protocols also result in some patients completing the survey by web who would have completed the survey by mail or phone if a web version was not available. Mail and phone survey administration is typically more expensive than web administration. After accounting for the fixed costs of designing and implementing the web-first mode, in the aggregate, substitution of a less expensive response mode (web) for more expensive response modes (mail and phone) suggests potential for cost savings. As the proportion of patients who provide email addresses increases, so will the potential RR gains and savings in data collection costs.

A randomized experiment can show relative differences in RRs by race and ethnicity for different survey methods in deidentified data such as HCAHPS. While RRs by race and ethnicity are not directly measurable, higher yields indicate higher RRs. For example, even though the proportion of sampled patients who are Black is unknown, randomization ensures that these proportions are similar across protocols. To calculate RRs by race and ethnicity, and not merely their ratio across protocols, would require a reliable administrative source of race and ethnicity data that included nonrespondents.

### Limitations

One limitation of this study is that because only hospitals with sufficient discharges for both the experiment and regular HCAHPS implementation could participate in the experiment, more than 90% of participating hospitals had at least 200 beds. While such hospitals are only 30% of all hospitals participating in HCAHPS, they nonetheless supplied 81.8% of all 2022 HCAHPS-eligible discharges (according to our calculations), and participating hospitals served patients similar to those from all HCAHPS hospitals except that their respondents were more diverse with respect to race and ethnicity, likely as a result of the inclusion of web-first modes. Finally, patients were randomized to survey protocols within hospitals to ensure comparability across modes.

Sequential multimode protocols increase RRs and representativeness by increasing the chance that patients will be offered their preferred mode, which varies across patients.^20^ The success of web-first protocols in increasing RRs for many underrepresented patient groups implies that many patients in these groups prefer responding by web to emailed survey invitations compared with other modes of survey completion.

## Conclusions

In this randomized clinical trial, adding web-first survey administration to any of the current official CMS HCAHPS survey protocols improved RRs and representativeness. Gains in RR from sequential web-first approaches were often 2 to 3 times as large for Asian American or Native Hawaiian or Other Pacific Islander, Black, Hispanic, and multiracial patients as the gain for White patients. In ancillary analysis, extending the HCAHPS response period from 42 to 49 days was also associated with greater representation of racially and ethnically underrepresented groups. While multimode approaches consistently outperform single-mode approaches, the most effective survey mode within a given hospital will depend on its specific patient population. Among 2-mode protocols, web-phone performed especially well for young and diverse patient populations, and web-mail for older and less diverse patient populations. Web-first survey protocols have the potential to improve HCAHPS RRs, reduce data collection costs, and support the evaluation of equity-targeted quality improvement efforts.^21^

While face-to-face surveys are still considered the criterion-standard survey administration mode when they are feasible,^22^ our results suggest that web-first multimode surveys may become the standard for some time to come in settings where in-person administration is infeasible but it is possible to collect email addresses. The email-based web approach used here avoids legal issues that vary by jurisdiction for outreaches by text.^23^ RRs from web alone are limited (10%-13%; only 16%-20% with complete EMA), so that follow-up modes, especially telephone mode, are likely to remain necessary to keep RRs and representativeness high across a variety of settings. The per-case costs associated with follow-up modes are only fully incurred when needed, which makes the web-first approach adaptable to different patient or other populations in national surveys. In light of these findings, CMS will allow hospitals to use the web-mail, web-phone, and web-mail-phone protocols for HCAHPS administration beginning in 2025.^24^

## Notes

Supplement 1. **eMethods.** Hospital Recruitment Strategy for the HCAHPS Mode Experiment and Sample Size Determination **eFigure.** Participant Flow for HCAHPS Mode Experiment **eTable 1. **Characteristics of the Hospitals, Sampled Eligible Patients, and Respondents **eTable 2.** Patient Characteristics of the Hospitals by Survey Administration Protocol, 46 Mode Experiment Hospitals **eTable 3.** Response Rates by Completion Mode and Survey Administration Protocol **eTable 4.** Standardized Response Rates by Mode and Age Group **eTable 5.** Standardized Response Rate by Mode and Service Line/Sex **eTable 6.** Yield and Increase in Yield Compared to Mail Only, by Age Group **eTable 7.** Yield and Increase in Yield Compared to Mail Only, by Service Line and Sex **eTable 8.** Characteristics of Early (0-42 days to survey completion) to Late (43-49 days to completion) Respondents

Supplement 2.
Data Sharing Statement
