# Error in Figure 2

**DOI:** 10.1001/jamahealthforum.2024.0186

**Published:** 2024-03-08

In the Original Investigation titled “Survey Protocols, Response Rates, and Representation of Underserved Patients: A Randomized Clinical Trial”^1^ published on January 19, 2024, there were errors in Figure 2. Panel B was replaced because it did not previously reflect the data meant to be displayed.
