# Supplementary material for: Survey Protocols, Response Rates, and Representation of Underserved Patients: A Randomized Clinical Trial
Source: JAMA Health Forum. 2024 Jan 19;5(1):e234929. doi: 10.1001/jamahealthforum.2023.4929 (PMC10799262; doi:10.1001/jamahealthforum.2023.4929)
Supplement: Supplement 1. — eMethods. Hospital Recruitment Strategy for the HCAHPS Mode Experiment and Sample Size Determination eFigure. Participant Flow for HCAHPS Mode Experiment eTable 1. Characteristics of the Hospitals, Sampled Eligible Patients, and Respondents eTable 2. Patient Characteristics of the Hospitals by Survey Administration Protocol, 46 Mode Experiment Hospitals eTable 3. Response Rates by Completion Mode and Survey Administration Protocol eTable 4. Standardized Response Rates by Mode and Age Group eTable 5. Standardized Response Rate by Mode and Service Line/Sex eTable 6. Yield and Increase in Yield Compared to Mail Only, by Age Group eTable 7. Yield and Increase in Yield Compared to Mail Only, by Service Line and Sex eTable 8. Characteristics of Early (0-42 days to survey completion) to Late (43-49 days to completion) Respondents [file jamahealthforum-e234929-s001.pdf]

Supplemental Online Content

Elliott MN, Brown JA, Hambarsoomian K, et al. Survey protocols, response rates, and representation of underserved patients. *JAMA Health Forum.* Published online January 19, 2024. doi:10.1001/jamahealthforum.2023.4929

eMethods. Hospital Recruitment Strategy for the HCAHPS Mode Experiment and Sample Size Determination

eFigure. Participant Flow for HCAHPS Mode Experiment

eTable 1. Characteristics of the Hospitals, Sampled Eligible Patients, and Respondents

eTable 2. Patient Characteristics of the Hospitals by Survey Administration Protocol, 46 Mode Experiment Hospitals

eTable 3. Response Rates by Completion Mode and Survey Administration Protocol

eTable 4. Standardized Response Rates by Mode and Age Group

eTable 5. Standardized Response Rate by Mode and Service Line/Sex

eTable 6. Yield and Increase in Yield Compared to Mail Only, by Age Group

eTable 7. Yield and Increase in Yield Compared to Mail Only, by Service Line and Sex

eTable 8. Characteristics of Early (0-42 days to survey completion) to Late (43-49 days to completion) Respondents

This supplemental material has been provided by the authors to give readers additional information about their work.

eMethods. Hospital Recruitment Strategy for the HCAHPS Mode Experiment and Sample Size Determination

Hospitals were sampled and enrolled in a three-stage process: In Stage 1, hospitals were randomly sampled within 12 strata. In Stage 2 (September 22, 2020- March 26, 2021), hospitals were contacted by mail and phone to confirm willingness to participate. In stage 3 (December 14, 2020- May 3, 2021), detailed questions about participation and data needs were addressed, and data use agreements were executed. We began with all hospitals that collected HCAHPS data in 2019 and have data reported on the CMS Hospital Compare Web site (now Care Compare). We then restricted to hospitals with sufficient HCAHPS-eligible discharges in 2019 to ensure that the hospitals would meet the minimum sample size requirements required for the mode experiment design after being able to collect at least 100 annual completed surveys for regular HCAHPS implementation. HVBP requires 100 annual completes, which corresponds to 50 completes over the 6 planned months of the mode experiment. A hospital was eligible if 75% of its remaining number of eligible surveys in 2019 was at least 535, after allocating enough surveys to obtain 50 completed surveys at its observed 2019 RR.

The 1,645 eligible hospitals, with 9,277,520 discharges (representing 81.8% of all eligible discharges) were classified into 12 strata using characteristics available in the American Hospital Association database, based on the factorial combinations of three characteristics: hospital bed size (< or =200 beds, >200 beds), HCAHPS HVBP score (below median, above median), and geographic region (Northeast, South, Midwest/West). Half of the hospitals were randomly sampled within each queue. Of the 823 hospitals, 170 hospitals (21%) agreed to participate in Stage 2. After learning more about the sampling and data requirements of the mode experiment, 88 hospitals (52% of 170, 11% of 823) agreed to review the data use agreement (DUA) necessary for sharing discharge data, and 46 hospitals (52% of 88, 27% of 170, 6% of 823) executed a DUA.

This design was expected to result in ~1,000 completes in each of the 12 combinations of 6 survey protocol arms and 2 email availability strata. under the anticipated response rates. For this design, an overall sample size of 41,714 eligible hospital patients were to be sampled over a six-month period; this sample size was obtained dividing 1000 by the projected response rate in each of the 12 design cells. Anticipated response rates were informed by the research literature, An overall 3% post-sampling ineligible rate was also assumed. See Participant Flow diagram below.

eFigure. Participant Flow for HCAHPS Mode Experiment

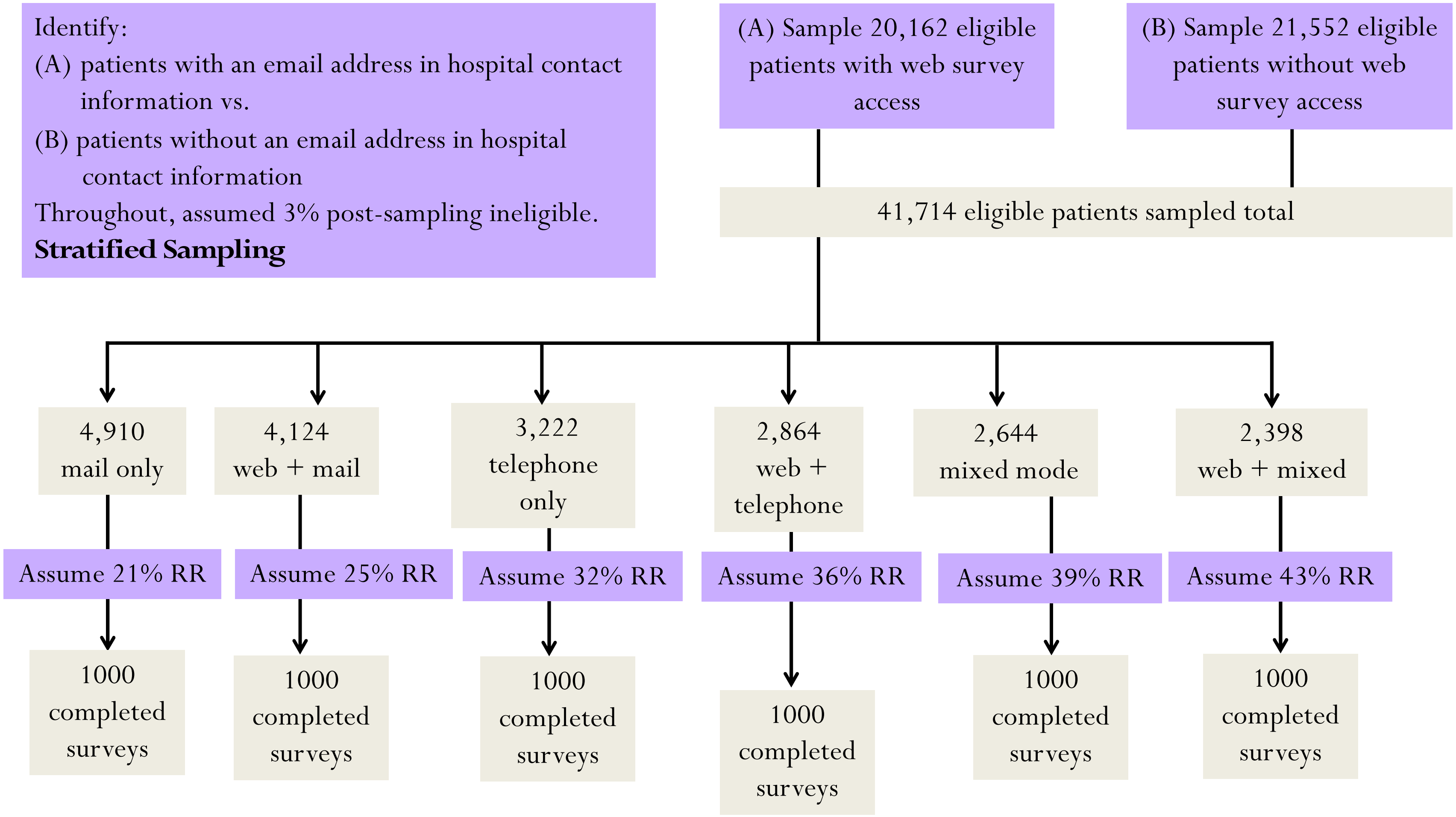

© 2024 Elliott MN et al. *JAMA Health Forum.*

Participant Flow for HCAHPS Mode Experiment

(cont.)

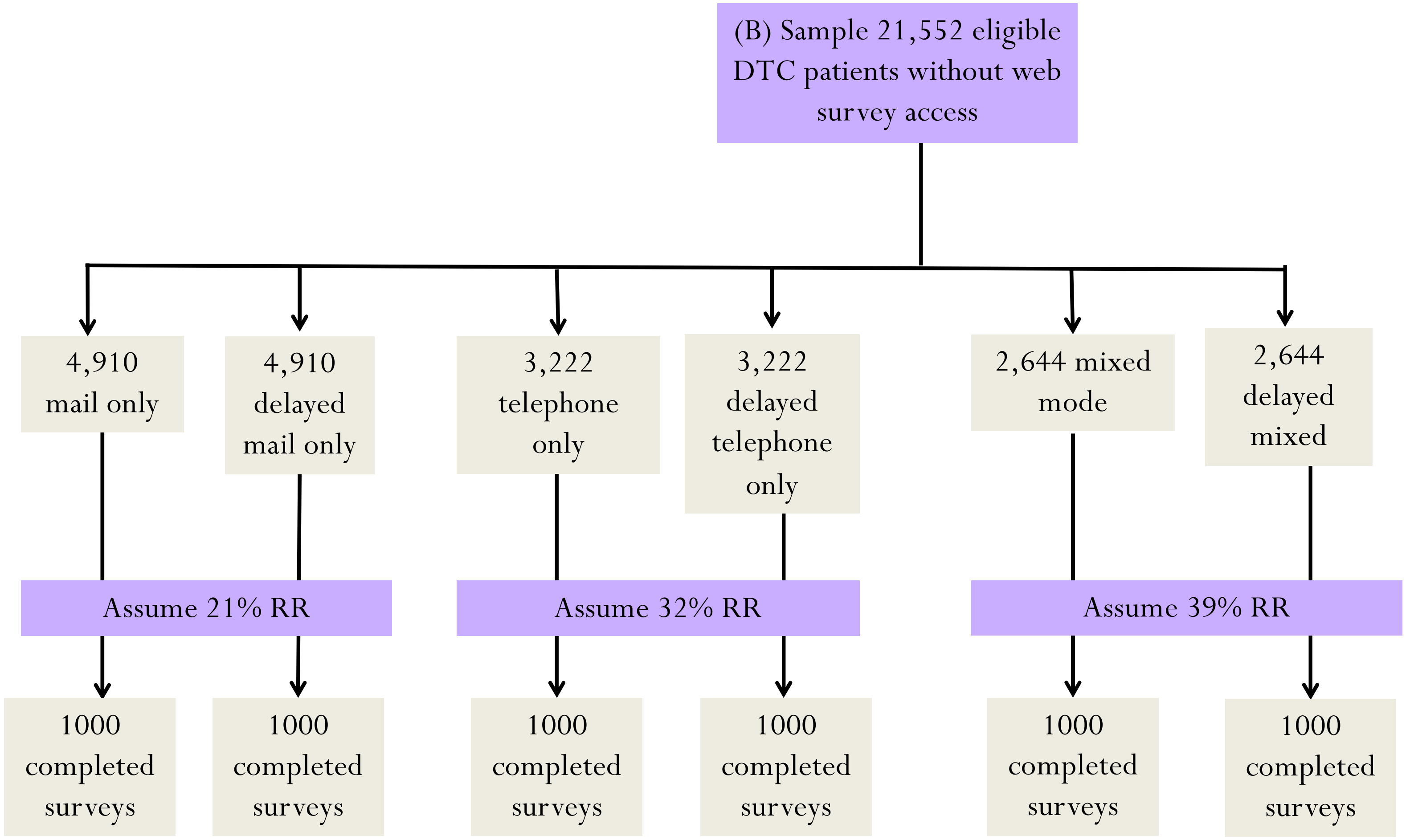

© 2024 Elliott MN et al. *JAMA Health Forum.*

eTable 1. Characteristics of the Hospitals, Sampled Eligible Patients, and Respondents

Characteristic	Mode experiment hospitals	2021 HCAHPS implementation hospitals
Hospitals	n = 46	n = 4556
Bed size		
<100	2.2%	51.6%
100 to 199	6.5%	19.3%
200 or more	91.3%	28.1%
Ownership		
Profit	26.1%	16.0%
Government/non-profit	73.9%	82.9%
Census regions		
Midwest	17.4%	29.7%
Northeast	10.9%	27.6%
South	45.7%	22.6%
West	26.1%	20.1%
HCAHPS HVBP[a]		
Above median	52.2%	50.0%
Below median	47.8%	50.0%
Sampled eligible patients	n = 34 335	n = 11 348 151
Age		
18-24	5.4%	5.9%
25-34	16.7%	14.9%
35-44	12.6%	10.9%
45-54	10.6%	11.2%
55-64	16.6%	17.5%
65-74	18.3%	19.1%
75-84	13.5%	13.9%
≥85	6.4%	6.6%
Service line × sex		
Maternity	19.2%	16.0%
Female medical	28.3%	30.4%
Male medical	26.9%	29.1%
Female surgical	12.5%	12.2%
Male surgical	13.2%	12.3%
Respondents	n = 9642	n = 2 301 815[1]
Race and ethnicity		
AA&NHPI[b]	6.9%	3.1%
AI/AN[c]	0.7%	0.7%
Black	11.5%	8.3%
Hispanic	17.4%	10.5%
White	61.0%	75.5%
Multiracial	2.6%	2.0%

© 2024 Elliott MN et al. *JAMA Health Forum.*

[a]Hospital Value-Based Purchasing.

[b]Asian American and Native Hawaii/Pacific Islander.

[c]American Indian and Alaska Native.

Percentages are reported excluding missing values from the denominator of ratio.

In the 2021 Mode Experiment Study, 19.0% (n = 6536) of the sampled eligible patients were missing service line and 3.5% (n = 339) of respondents were missing race-and-ethnicity.

In the 2021 Implementation Data, 5.4% (n = 124 298) of respondents from all hospitals were missing race-and-ethnicity.

HCAHPS HVBP scores are based on the 2927 hospitals for which an HVBP was calculated (including all 46 mode experiment hospitals).

© 2024 Elliott MN et al. *JAMA Health Forum.*

eTable 2. Patient Characteristics of the Hospitals by Survey Administration Protocol, 46 Mode Experiment Hospitals

	Mail-Only	Phone-Only	Mail-Phone	Web-Mail	Web-Phone	Web-Mail-Phone
**Sampled eligible patients (n=34,335)**						
Age						
18-24	5.1%	5.4%	5.2%	5.4%	5.5%	5.9%
25-34	15.9%	17.1%	17.4%	15.9%	17.0%	17.4%
35-44	12.7%	12.8%	12.8%	12.0%	13.1%	12.2%
45-54	10.6%	10.9%	10.5%	10.9%	10.2%	10.5%
55-64	17.0%	16.6%	16.6%	16.3%	16.9%	16.1%
65-74	18.2%	18.0%	18.1%	19.2%	18.0%	17.8%
75-84	13.6%	13.2%	13.2%	13.7%	13.6%	13.7%
85+	7.0%	6.0%	6.3%	6.6%	5.8%	6.4%
Service line x Sex						
Maternity	18.5%	19.7%	20.0%	18.5%	19.1%	19.9%
Female Medical	29.1%	27.9%	28.2%	27.8%	28.4%	28.4%
Male Medical	27.1%	26.8%	26.1%	27.0%	26.6%	27.5%
Female Surgical	12.8%	12.4%	12.8%	12.7%	12.3%	11.8%
Male Surgical	12.6%	13.2%	12.9%	14.1%	13.6%	12.4%
**Respondents (n=9,642)**						
Race-and-ethnicity						
AA&NHPI[a]	5.8%	6.4%	7.9%	8.0%	6.8%	6.2%
AI/AN[b]	0.8%	0.4%	0.9%	0.6%	0.8%	0.7%
Black	10.7%	14.1%	11.3%	9.6%	11.4%	12.9%
Hispanic	13.6%	20.8%	16.1%	16.8%	20.2%	17.5%
White	68.6%	52.7%	60.5%	63.8%	56.8%	61.0%
Multiracial	0.7%	5.6%	3.3%	1.2%	4.0%	1.7%

[a]Asian American and Native Hawaii/Pacific Islander

[b]American Indian and Alaska Native

© 2024 Elliott MN et al. *JAMA Health Forum.*

eTable 3. Response Rates by Completion Mode and Survey Administration Protocol

Survey Administration Protocol	Completion Mode	Pooled	EMA	No EMA
Mail-Only	Mail	22.8%	23.7%	21.2%
Phone-Only	Phone	22.1%	23.1%	20.3%
Mail-Phone	Mail	17.7%	19.1%	15.3%
	Phone	13.5%	15.0%	10.8%
Web-Mail	Web	12.6%	19.9%	N/A
	Mail	16.1%	13.6%	20.3%
Web-Phone	Web	13.0%	20.3%	N/A
	Phone	17.5%	16.8%	18.7%
Web-Mail-Phone	Web	10.1%	15.7%	N/A
	Mail	13.1%	11.5%	16.0%
	Phone	13.0%	12.8%	13.3%

© 2024 Elliott MN et al. *JAMA Health Forum.*

eTable 4. Standardized Response Rates by Mode and Age Group

Age	Mail-Only (ref)	Phone-Only	Mail-Phone	Web-Mail	Web-Phone	Web-Mail-Phone
**18-24**	8.5% (5.6%, 11.5%)	19.6%* (15.0%, 24.1%)	20.8%* (15.7%, 25.9%)	11.0% (7.8%, 14.2%)	**26.8%*** (21.8%, 31.8%)	21.1%* (16.3%, 25.9%)
**25-34**	10.0% (8.2%, 11.8%)	17.4%* (15.0%, 19.8%)	23.2% * (20.2%, 26.1%)	23.7%* (21.1%, 26.2%)	27.4%* (24.5%, 30.2%)	**30.2%*** (27.1%, 33.3%)
**35-44**	13.9% (11.6%, 16.2%)	20.8%* (17.8%, 23.8%)	25.5%* (22%, 29%)	21.1%* (18.3%, 24%)	27.0%* (23.8%, 30.3%)	**28.5%*** (24.8%, 32.2%)
**45-54**	16.3% (13.6%, 19.1%)	22.5%* (19.2%, 25.8%)	22.3%* (18.6%, 26%)	20.0% (17.0%, 22.9%)	28.4%* (24.7%, 32.1%)	**31.9%*** (27.7%, 36.1%)
**55-64**	23.8% (21.2%, 26.3%)	22.9% (20.2%, 25.6%)	32.2%* (28.9%, 35.4%)	29.7%* (27.0%, 32.5%)	32.6%* (29.5%, 35.6%)	**39.5%*** (36%, 43%)
**65-74**	37.4% (34.6%, 40.2%)	25.7%* (23.0%, 28.4%)	39.0% (35.7%, 42.3%)	41.7%* (38.8%, 44.5%)	32.4%* (29.5%, 35.2%)	**43.7%*** (40.3%, 47%)
**75-84**	40.4% (37.1%, 43.8%)	25.4%* (22.3%, 28.5%)	43.6% (39.7%, 47.6%)	46.0%* (42.6%, 49.5%)	35.3% (31.9%, 38.7%)	**47.2%*** (43.2%, 51.2%)
**85+**	40.8% (35.7%, 45.8%)	19.7%* (15.4%, 24%)	36.4% (30.9%, 41.9%)	**41.0%** (35.8%, 46.2%)	27.9%* (23.2%, 32.6%)	40.5% (34.8%, 46.2%)

*$p<0.05$ for difference from Mail-Only via logistic regression. Confidence intervals are based on a normal approximation of the binomial.

Bold is highest response rate for a given row.

© 2024 Elliott MN et al. *JAMA Health Forum.*

eTable 5. Standardized Response Rate by Mode and Service Line/Sex

	Mail-Only (ref)	Phone-Only	Mail-Phone	Web-Mail	Web-Phone	Web-Mail-Phone
**Maternity**	12.3% (10.3%, 14.4%)	18.6%* (16%, 21.2%)	26.8%* (23.7%, 30.0%)	28.1%* (25.3%, 30.9%)	**32.2%*** (29.1%, 35.3%)	**32.2%*** (28.9%, 35.6%)
**Female, Medical**	24.5% (22.3%, 26.7%)	19.4%* (17.2%, 21.5%)	29.7%* (27.0%, 32.5%)	27.9% (25.6%, 30.3%)	26.8% (24.4%, 29.2%)	**37.7%*** (34.7%, 40.6%)
**Male, Medical**	23.7% (21.5%, 26.0%)	22.0% (19.6%, 24.3%)	28.7%* (25.9%, 31.5%)	27.8%* (25.4%, 30.2%)	27.6%* (25.1%, 30.1%)	**33.9%*** (31%, 36.8%)
**Female, Surgical**	35.0% (31.4%, 38.7%)	28.7%* (25.0%, 32.5%)	41.2% (36.7%, 45.6%)	41.9%* (38.2%, 45.7%)	36.6% (32.6%, 40.6%)	**43.2%** (38.6%, 47.8%)
**Male, Surgical**	31.1% (27.5%, 34.6%)	27.2% (23.6%, 30.8%)	37.7%* (33.4%, 42.1%)	35.9%* (32.4%, 39.4%)	33.7% (29.9%, 37.4%)	**41.3%*** (36.8%, 45.7%)

*$p<0.05$ for difference from Mail-Only via logistic regression. Confidence intervals are based on a normal approximation of the binomial.

Bold is highest response rate for a given row.

© 2024 Elliott MN et al. *JAMA Health Forum.*

eTable 6. Yield and Increase in Yield Compared to Mail Only, by Age Group

	Yield	Percentage Increase in Yield Compared to Mail-Only (95% Confidence Interval) Mail-Only				
**Age**	Mail-Only (ref)	Phone-Only	Mail-Phone	Web-Mail	Web-Phone	Web-Mail-Phone
**18-24**	0.4<br>(0.3, 0.5)	+150%*<br>(87%, 213%)	+150%*<br>(81%, 219%)	+50%<br>(5%, 95%)	**250%***<br>(176%, 324%)	+200%*<br>(125%, 275%)
**25-34**	1.5<br>(1.2, 1.8)	+87%*<br>(59%, 115%)	+147%*<br>(112%, 182%)	+140%*<br>(111%, 169%)	+187%*<br>(152%, 221%)	**+233%***<br>(193%, 274%)
**35-44**	1.7<br>(1.4, 2.0)	+47%*<br>(24%, 70%)	+82%*<br>(54%, 111%)	+41%*<br>(20%, 62%)	**+94%***<br>(68%, 121%)	**+94%***<br>(65%, 123%)
**45-54**	1.6<br>(1.3, 1.9)	+44%*<br>(20%, 67%)	+38%*<br>(12%, 63%)	+25%<br>(5%, 45%)	+69%*<br>(43%, 94%)	**+94%***<br>(64%, 124%)
**55-64**	3.8<br>(3.4, 4.2)	-5%<br>(-18%, 7%)	+32%*<br>(16%, 47%)	+18%*<br>(6%, 31%)	+34%*<br>(20%, 49%)	**+55%***<br>(38%, 72%)
**65-74**	6.3<br>(5.7, 6.9)	-32%*<br>(-40%, -24%)	+3%<br>(-8%, 14%)	**+17%***<br>(8%, 27%)	-14%*<br>(-23%, -5%)	+16%*<br>(4%, 27%)
**75-84**	4.9<br>(4.4, 5.4)	-35%*<br>(-44%, -26%)	+10%<br>(-3%, 23%)	+16%*<br>(5%, 27%)	-8%<br>(-19%, 3%)	**+20%***<br>(7%, 34%)
**85+**	**2.4**<br>(2.0, 2.8)	-54%*<br>(-65%, -43%)	-13%<br>(-29%, 4%)	-8%<br>(-23%, 6%)	-33%*<br>(-47%, -20%)	**0%**<br>(-18%, 18%)

*p<0.05 for difference from Mail-Only via logistic regression. Confidence intervals are based on a normal approximation of the binomial.

Bold is highest response rate for a given row.

Yield is the ratio of number of completes by mode and age divided by the number fielded within a protocol

© 2024 Elliott MN et al. *JAMA Health Forum.*

eTable 7. Yield and Increase in Yield Compared to Mail Only, by Service Line and Sex

	Yield	Percentage Increase in Yield Compared to Mail-Only (95% Confidence Interval) Mail-Only				
	Mail-Only (ref)	Phone-Only	Mail-Phone	Web-Mail	Web-Phone	Web-Mail-Phone
**Maternity**	1.8<br>(1.5, 2.1)	+50%*<br>(27%, 73%)	+122%*<br>(92%, 152%)	+128%*<br>(102%, 154%)	+161%*<br>(131%, 191%)	**+172%***<br>(139%, 205%)
**Medical, female**	5.3<br>(4.8, 5.8)	-23%*<br>(-32%, -13%)	+17%*<br>(4%, 30%)	+8%<br>(-3%, 18%)	+9%<br>(-2%, 21%)	**+51%***<br>(37%, 65%)
**Medical, male**	4.8<br>(4.3, 5.3)	-8%<br>(-19%, 2%)	+19%*<br>(5%, 32%)	+17%*<br>(5%, 28%)	+15%*<br>(3%, 27%)	**+44%***<br>(29%, 58%)
**Surgical, female**	3.4<br>(3.0, 3.8)	**-21%***<br>(-33%, -9%)	+15%<br>(-1%, 30%)	**+21%***<br>(7%, 34%)	+0%<br>(-13%, 13%)	+15%<br>(-1%, 30%)
**Surgical, male**	3.0<br>(2.6, 3.4)	-10%<br>(-24%, 4%)	+20%*<br>(3%, 37%)	+27%*<br>(12%, 42%)	+13%<br>(-2%, 29%)	**+30%***<br>(12%, 48%)

*p<0.05 for difference from Mail-Only via logistic regression. Confidence intervals are based on a normal approximation of the binomial.

Bold is highest response rate for a given row.

© 2024 Elliott MN et al. *JAMA Health Forum.*

eTable 8. Characteristics of Early (0-42 days to survey completion) to Late (43-49 days to completion) Respondents

	Percentage			Test (Early vs Late)
	Early	Late	Late - Early	p-value
	n=8,392 (87%)	n=1,250 (13%)		
Sex				
Female	59%	60%	1%	0.40
Age				
18-24	3%	3%	0%	0.99
25-34	12%	14%	2%	0.04
35-44	10%	10%	0%	0.59
45-54	9%	8%	0%	0.83
55-64	17%	19%	2%	0.07
65-74	23%	21%	-2%	0.14
75-84	19%	16%	-3%	0.03
85+	7%	7%	0%	0.97
Language Spoken at Home				
English	90%	87%	-4%	0.000
Spanish	5%	7%	2%	0.01
Chinese	1%	1%	0%	0.25
Other	4%	6%	2%	0.005
Education				
8th grade	3%	4%	1%	0.01
Some high school	6%	8%	1%	0.09
High school	27%	28%	2%	0.19
Some college	31%	32%	1%	0.56
4-year college degree	16%	13%	-3%	0.005

© 2024 Elliott MN et al. *JAMA Health Forum.*

More college	17%	15%	-2%	0.08
Sex x Service line				
Female medical	27%	29%	2%	0.08
Female surgical	17%	15%	-2%	0.07
Maternity	16%	17%	1%	0.19
Male medical	25%	24%	-1%	0.44
Male surgical	16%	15%	-1%	0.45
Overall Health				
Excellent	13%	12%	-1%	0.50
Very good	25%	23%	-2%	0.25
Good	33%	33%	0%	0.85
Fair	22%	23%	1%	0.34
Poor	7%	9%	1%	0.11
Mental Health				
Excellent	27%	25%	-2%	0.15
Very good	33%	32%	-1%	0.47
Good	27%	28%	1%	0.41
Fair	12%	13%	1%	0.42
Poor	2%	3%	1%	0.02
Race/Ethnicity				
American Indian/Alaska Native	1%	0%	0%	0.21
Asian American & Native Hawaiian or Pacific Islander	6%	8%	1%	0.11
Black	11%	13%	3%	0.005
Hispanic	17%	18%	2%	0.09
Multiracial	2%	3%	0%	0.56
White	60%	49%	-11%	0.000
Unknown	3%	8%	5%	0.000
Hospital Census Division				
New England	3%	3%	0%	0.63
Pacific	26%	24%	-2%	0.10

© 2024 Elliott MN et al. *JAMA Health Forum.*

East North Central	12%	10%	-2%	0.04
East South Central	11%	11%	0%	0.98
Mid-Atlantic	8%	11%	3%	0.001
Mountain	10%	9%	0%	0.66
South Atlantic	16%	17%	1%	0.37
West North Central	5%	5%	-1%	0.40
West South Central	9%	10%	1%	0.17
Hospital Bed Size				
<100	2%	1%	-1%	0.10
100-199	6%	5%	-1%	0.26
200+	93%	94%	1%	0.07
Hospital Ownership				
For-profit	23%	25%	2%	0.16
Teaching Hospital	70%	71%	2%	0.21
Rural Hospital	2%	1%	-1%	0.03

P-values are estimated using logistic regression.

© 2024 Elliott MN et al. *JAMA Health Forum.*
