# Supplementary material for: Survey Protocols, Response Rates, and Representation of Underserved Patients: A Randomized Clinical Trial
Source: JAMA Health Forum. 2024 Jan 19;5(1):e234929. doi: 10.1001/jamahealthforum.2023.4929 (PMC10799262; doi:10.1001/jamahealthforum.2023.4929)
Supplement: Supplement 2. — Data Sharing Statement [file jamahealthforum-e234929-s002.pdf]

Data Sharing Statement

Elliott. Survey Protocols, Response Rates, and Representation of Underserved Patients. *JAMA Health Forum*. Published January 19, 2024. doi:10.1001/jamahealthforum.2023.4929

Data

Data available: No

Additional Information

Explanation for why data not available: Data cannot be shared publicly because it is proprietary, institutional data. Data are available from the Centers for Medicare and Medicaid Services for researchers who meet the criteria for access to confidential data.
